# Health lifestyles and Chinese oldest-old’s subjective well-being—evidence from a latent class analysis

**DOI:** 10.1186/s12877-021-02121-0

**Published:** 2021-03-24

**Authors:** Li Zhang, Xiangyang Bi, Zhihong Ding

**Affiliations:** 1 School of Sociology, China University of Political Science and Law, Beijing, 102249 China; 2 School of Ethnology and Sociology, Minzu University of China, No. 27, Zhongguancunnan Street, Haidian District, Beijing, 100081 China; 3 School of Sociology and Psychology, Central University of Finance and Economics, Beijing, 100081 China

**Keywords:** Health lifestyles, Socioeconomic status, Latent class analysis, Chinese oldest-old, CLHLS, Subjective well-being

## Abstract

**Background:**

Previous research on the associations between lifestyle behaviors and health has largely focused on morbidity, mortality and disease prevention. More attention should be paid to examining relationships between lifestyle behaviors and positive health outcomes such as well-being. The aim of the study was to classify Chinese oldest-old’s health lifestyles and evaluate the manner in which health lifestyles have impacted Chinese oldest-old’s subjective well-being.

**Methods:**

Analyzing the 2014 Chinese Longitudinal Healthy Longevity Survey (CLHLS), latent class analysis was applied to identify predominant health lifestyles among Chinese oldest-old aged 85 to 105. Ordinary Least Square (OLS) regression models were used to assess the effects of health lifestyles on Chinese oldest-old’s subjective well-being, adjusting for socio-demographic characteristics.

**Results:**

Four distinct classes representing health lifestyles emerged. Health lifestyles were found to be strongly associated with Chinese oldest-old’s subjective well-being, even after controlling for demographic features as well as individual and parental socioeconomic disadvantage. Findings showed that healthy lifestyle behaviors stimulated Chinese oldest-old’s positive feelings and led to better evaluative subjective well-being. In contrast, less healthy lifestyle behaviors can be a predictor of negative feelings.

**Conclusions:**

The regression results highlighted the importance of integrating health lifestyle choices in promoting oldest-old’s psychological well-being. Elders can tackle healthier lifestyle behaviors in their daily lives to reduce the risk of mental health problems. Practicing healthy lifestyles should be integrated in programs for mental health promotion.

**Supplementary Information:**

The online version contains supplementary material available at 10.1186/s12877-021-02121-0.

## Background

Physical and mental health disparities caused by a variety of behaviors have been well studied by the existing literature. Individual or single health behaviors that have been commonly examined in prior analyses included physical activities, sleeping, poor dietary habits, cigarette smoking, excessive alcohol consumption et al. [1–7]. In recent years, however, researchers have begun to use clustered health lifestyle approach to explain health disparities among individuals. The benefit of the health lifestyle perspective is that it has extended the scopes of previous analyses on single health behaviors to classified health lifestyles [8]. Health lifestyles tended to cluster in ways that reflect social and structural contexts of individuals, which in turn affects individual health status, including subjective well-being [9, 10]. Thus, considering multiple behaviors simultaneously is believed to be a more appropriate strategy that creates larger and more enduring behavior change to improve individual health and well-being [11].

Under such a proceeding, when explaining health disparities, including mental health and subjective well-being, recent studies have shifted their interests to investigating health lifestyles behaviors and a strong association has been found in both ways. First, subjective well-being was documented to have a protective effect on maintaining healthy lifestyle behaviors [12]. Scholars emphasized that people with higher positive affect, a component of subjective well-being, were more likely to exercise and not smoking [13]. Young adults who reported a higher level of life satisfaction were also more prone to participate in physical activities, eat more fruits and vegetables, consume less tobacco and alcohol, and use sun protection [14, 15]. Clinical studies further proved such a strong connection between subjective well-being and health lifestyles. For instance, among patients with heart diseases, those with higher positive affect at baseline were found to have a higher likelihood of practicing healthier behaviors [16]. Individuals who had higher positive affect also had higher odds of survival at follow-up [17].

Meanwhile, empirical analyses supported a significant positive effect of health lifestyle behaviors on individual subjective well-being [18]. Prior research documented that being physically active and having lunch, fruits and vegetables daily were positively associated with greater happiness [19]. Longitudinal studies showed that physical activity and lifelong physical exercise can prevent depressive symptoms and maintain positive self-esteem in older people [20]. Through investigating Spanish elderly sample, physical activity and physical function were demonstrated to enhance one’s well-being [21]. A study of Chinese sample showed that regular physical examinations improved individual life satisfaction, an important component of subjective well-being [22]. Engaging in physical exercises, such as sport game attendance, was found to increase older adults’ emotional support, their sense of belonging and subjective well-being [23]. Recent studies on individuals in News land found that exercise and high quality of sleep, were associated with individual well-being [24, 25]. Lower-risk health lifestyle behaviors were also documented to be positively associated with excellent well-being among Australians, after adjusting for socio-demographics, chronic diseases, depression and anxiety [26]. A significantly positive effect of healthy lifestyle behaviors on subjective well-being was discovered among Spain sample aged 65 and over as well [27].

The existing studies have largely advanced our understanding on health lifestyles and individual subjective well-being. Nonetheless, few studies concentrated on exploring the link between health lifestyles and Chinese oldest-old’s subjective well-being. Indeed, most existing analyses on Chinese older adults focused on the effects of single health behaviors on Chinese older adults’ health status and subjective well-being [28–31]. With the increasing life expectancy of Chinese population, it becomes critical to investigate how elderly Chinese could maintain a good quality of life and live healthier. Exploring potential factors that may improve the oldest-old’s health status and subjective well-being may well be an effective way to alleviate the burden of the society as well as family caregivers. This study, therefore, relied on the latent class analysis strategy to analyze data from the 2014 wave of the Chinese Longitudinal Health and Longevity Survey (CLHLS), a nationally representative survey, to include health behaviors from multiple domains and present a relatively more comprehensive picture of health behaviors among Chinese oldest-old. It also aimed to elucidate how health lifestyles have shaped Chinese oldest-old’s subjective well-being. Findings based on scrutinizing the nationally representative data in China are valuable to address disease prevention and health promotion related issues among the oldest-old in world countries. Exploring how clustered health behaviors influence Chinese oldest-old’s health outcomes can also expand theories explaining health disparities among older adults in general. Below the paper moved to an introduction of data, measures and methods used in the study.

## Data and Measures

### Data

Data came from the 2014 Chinese Longitudinal Healthy Longevity Survey (CLHLS) which was conducted in randomly selected half of the counties/cities in 22 provinces of China. Until now, 7 waves (1998, 2000, 2002, 2005, 2008, 2011–12, and 2014) of survey data have been collected. The survey was initially launched to meet the needs for scientific research on the oldest-old. Thus, the dataset provided an excellent source for studying the oldest-old in China. Previous literature showed that persons who reported age 106 or higher were considered as invalid cases [32]. Thus, persons aged 106 and higher were excluded from this study due to insufficient information to validate their reported extremely high age. The study eventually obtained 3416oldest-old aged 85 to 105, with 2025males and 1391females.

### Measures

#### Health lifestyle indicators

The selection of health lifestyle indicators in this research has been largely guided by prior studies. Health lifestyles measures used in previous analyses can be classified as the following categories: (1) dietary patterns (including eating fruits, vegetables, breakfast et al.), (2) smoking, alcohol consumption, (3) sleep, (4) obesity and physical activity, (5) seat belt wearing and media use, (6) body mass index (BMI), and (7) regular physical examination [12, 33–39]. Due to the availability of measures in the CLHLS dataset, four key sets of health lifestyle measures were applied, including dietary behaviors, smoking and alcohol use, sleep, and physical and leisure activities.

The first set was dietary behaviors. In the CLHLS survey, the respondent was asked the frequency of eating or drinking fresh fruit, fresh vegetables and tea. The study coded these three variables as dichotomous ones with labeling respondents answering “almost everyday” and as “1” and “0” if otherwise. Tea consumption was considered because previous research pointed out that tea drinking related to longevity and reduced risk of mortality and death from cardiovascular diseases [40]. Tea consumption was thus used as an important health lifestyle behavior in this study.

The second set of health lifestyle measures related to smoking and alcohol use. Since the variables measuring the respondent’s exact amount of cigarette or alcohol consumption had an extremely large amount of missing values with responding rates lower than 20.0% of the total sample, the research applied other measures. Those measures relied on CLHLS survey questions asking the respondent whether he or she smoked or drank alcohol “in the past” and “at present”. The respondent who never smoked in the past or at present was coded as “0” and “1” if otherwise. It was assumed that for those individuals who smoked in the past and was still smoking when the survey was conducted was a heavy smoker; the same rationale and coding strategy were also applied to the alcohol consumption variable.

Sleep was the third set of health lifestyle measures which was represented by two indicators: sleeping duration and sleep quality. The study dichotomized the sleep duration variable as “1” indicating having 8 h or more sleep each day and “0” as having less than 8 h sleep. The sleep quality variable was dichotomized with those who reported their sleep quality as “good” and “very good” as “1” and poor sleep quality as”0″ (including the categories that were originally coded in the survey as ‘so so’, ‘bad’ and ‘very bad’).

The fourth set was physical and leisure activities. The research relied on two survey questions asking whether the respondent exercised regularly in the past and at present to determine if he or she was physically active. Those who exercised regularly both at present and during the past were coded as “1”, and “0” if otherwise. The research also classified leisure activities into sedentary and active activities. Sedentary activities were such as reading newspapers/books, playing cards and /or mah-jong, and watching TV and/or listening to radio. Active activities included raising domestic animals, doing gardening work et al. For those who participated in leisure activities almost everyday were coded as “1” and “0” if otherwise. Thus, healthy lifestyle measures included in the study can be listed as follows:

1. Eating fresh fruits almost everyday (No/Yes)
2. Eating fresh vegetables almost everyday (No/Yes)
3. Drinking tea almost everyday (No/Yes)
4. Smoked in the past and was still smoking (No/Yes)
5. Drank in the past and was still smoking (No/Yes)
6. Had 8 h or more sleep each day (No/Yes)
7. Described sleep quality as “good” and “very good”(No/Yes)
8. Exercised regularly both at present and during the past (No/Yes)
9. Participated in sedentary leisure activities almost everyday (No/Yes)
10. Participated in active leisure activities almost everyday (No/Yes)

#### Subjective well-being measures

Subjective well-being includes an individual’s satisfaction with various domains of life, which can be considered as a person’s overall judgment of life satisfaction and his/her effective state measured by the amount of negative or positive emotions [41]. Such a definition of subjective well-being generally contains two components which are *experienced* and *evaluative* subjective well-being. *Experienced* well-being means positive and negative emotions that an individual experiences in daily life. *Evaluative* well-being is more related to cognitive judgment of one’s life satisfaction on various domains, including marriage, job, family et al. [42].

The subjective well-being measures used in this research were consistent with measures used in previous research, which was operationalized by a series of CLHLS questions evaluating the elderly’s life. The questions used to measure *experienced well-being* were as follows: 1) Do you always look on the bright side of things? 2) Are you happy now as when you were younger? 3) Do you often feel fearful or anxious? 4) Do you often feel lonely and isolated? 5) Do you feel the older you get the more useless you are? The responses ranged from 1 to 5. “1” represents always or very good; “5” represents never or very bad. The items were recoded so that “1” indicated the weakest feeling and “5” the strongest feeling. Since CLHLS data were not collected to examine the psychological well-being of the elderly, the above questions may not be perfect indicators of one’s subjective well-being. However, Chen and Short [43] pointed out that these measures represented important dimensions of subjective well-being, such as life satisfaction, happiness and loneness. Thus, the research considered measures associated with the above questions as legitimate indicators of older adults’ subjective well-being.

This study generated two indices by adding items 1 to 2 as an index representing positive well-being, adding items 3 to 5 as the index of negative well-being. For the positive well-being measure, it ranged from 2 to 10, with higher numbers indicating better well-being. The value for the index of negative well-being ranged from 3 to 15, with higher values indicating worse well-being. The above questions were considered as independent to each other. And the indices were created by adding the raw scores of all variables in each set and were summary scores for each set of variables discussed above. The logic of constructing the above indices was that each group of variables measured the same concept. This strategy reduced the number of variables in the analysis and improved the efficiency of the regression models estimating the relationship between health lifestyles and the oldest-old’s subjective well-being. After summing each set of variables to a single variable, Cronbach’s alpha was used to assess the reliability of a given set of variables. The reliability alpha (Cronbach’s alpha) was defined as the square of the correlation between the measured scale and the underlying factor. The value of alpha represented the expected correlation of one test with an alternative form containing a number of items. The statistical results showed that the internal consistency coefficients for the indices were alpha =0.51 and 0.63, respectively. The two alpha values seemed to be lower than the alpha value used in other research. But since only two or three items were used to construct the indices and the alpha value is positively related to number of items used, the alpha values were considered as acceptable.

The *evaluative well-being* was measured by the question: “Overall, how do you rate your present life?” The coding scale was a 5-point scale, that is, 5 = very good, 4 = good, 3 = ‘so so’ (in effect, ‘neither good nor bad’), 2 = bad and 1 = very bad. Life satisfaction reflects quality of life from the subjective feelings of persons, which is also considered as an overall evaluation of one’s quality of life. The Appendix specified the alpha values and the items which were used to compose subjective well-being variables in the analysis.

#### Control variables

The analysis also controlled for the respondent’s demographic characteristics such as age, gender, rural and urban residence. Respondents who lived in cities and towns were classified as urban residents. The respondent’s socioeconomic status (SES) was also controlled, including years of schooling, per capita household income, and occupation before age 60. The occupation variable was coded as “1” if the respondent held a professional or administrative occupation and “0” if otherwise. Since socioeconomic condition in early childhood has been documented to have a cumulative effect on one’s later life health status [44, 45], “whether the respondent frequently went to bed hungry as a child” was also controlled as a measure of the early childhood (or parental) SES. The study also controlled for whether the respondent’s spouse died in the past 3 years because this event has been found to be significantly related to an individual’s subjective well-being [43]. Widowhood was also controlled because widowed older adults were documented to be lonelier than married ones and their subjective well-being may be poor [46]. The analysis also controlled for the oldest-old’s living arrangements (if living alone = 1, 0 if otherwise), self-rated health (SRH) and activity of daily living (ADL) disabilities (1 = yes, 0 = no) since those factors have been found to be influential on one’s subjective well-being [47]. Table 1 showed descriptive statistics for all variables.

**Table 1:** Summary Statistics for All Variables: Chinese Oldest-Old Aged 85–105 (*N* = 3416)

Variables	Mean (or %)	SD	N
*Health lifestyle variables*			
1) If R eats fresh fruit almost everyday			3349
Yes	13.5		
No	86.5		
2) If R eats fresh vegetables almost everyday			
Yes	49.1		3347
No	50.9		
3) If R drinks tea almost everyday			
Yes	17.2		3321
No	82.8		
4) If R smoked before and is still a smoker			3416
Yes	24.9		
No	75.1		
5) If R drank before and is still a drinker			3416
Yes	21.3		
No	78.7		
6) R’s quality of sleep			3347
Good	60.4		
Poor	39.1		
7) If R normally sleeps at least 8 h			3314
Yes	55.3		
No	44.7		
8) If R exercised during the past and still exercises at present			3416
Yes	26.8		
No	73.2		
9) If R participates active leisure activities			3416
Yes	48.4		
No	51.6		
10) If R participates sedentary leisure activities			3416
Yes	41.7		
No	58.3		
*Subjective well-being variables*			
Self-rated life	3.8	0.7	2979
Positive feelings index	7.2	1.6	2616
Negative feelings index	7.0	2.3	2715
**Control variables**			
* R’s characteristics*			
Age (mean)	93.1	5.7	3416
Gender (male =1)			3416
Male	40.7		
Female	59.3		
Rural/urban residence (urban =1)			3416
Urban	42.7		
Rural	57.3		
R’s reported years of schooling (mean)	1.5	2.8	3364
R’s household per capita income	15,832.8	17,233.8	2388
R’s had professional or administrative job before age 60			3115
Yes	5.7		
No	94.3		
R’s self-rated health (mean)	3.3	0.9	2977
If R has ADL disability			3416
Yes	34.8		
No	65.2		
If R’s spouse died in past three years			2387
Yes	21.3		
No	78.7		
If R lived alone			3334
Yes	19.8		
No	80.2		
* R’s Parental characteristics*			
Whether R often went to bed hungry in childhood			2977
Yes	77.4		
No	22.6		

*Notes:* Some sub-categories may not add up to 100% due to rounding. R represents respondent

*Source*: Chinese Longitudinal Healthy Longevity Survey (CLHLS) 2014 data

## Methods

### Latent class analysis

Latent class analysis (LCA) was used to predict membership in latent or unobserved groups. LCA is a statistical method that finds subtypes of related cases (latent classes) from multivariate categorical data. It can be used to find distinct categories considering presence/absence of several symptoms. The rationale of LCA is that given a sample of cases (subjects, objects, respondents, patients, et al.) measured on several variables, one wishes to know if there is a small number of basic groups into which cases fall. The results of LCA can be used to classify cases to their most likely latent class. Within each latent class, each variable is statistically independent of every other variable. If one removes the effect of latent class membership on the data, all that remains is randomness. In this study, respondents in a certain latent class shared similar health lifestyle patterns, and each case was also assigned a probability of membership in each class.

LCA uses observed data to estimate parameter values for the model. The model parameters are the prevalence of each of C case subpopulations or latent classes and conditional response probabilities. A randomly selected member of that class will make that response to that item/variable. Parameters are estimated by the maximum likelihood (ML) criterion. The ML estimates are those most likely to account for the observed results. Estimation is usually done with simple iterative numerical methods. The traditional forms of LCA used complicated estimation methods based on matrix manipulation and simultaneous linear equations. Later on, simple iterative proportional fitting was used to find ML parameter values. In this analysis, since the exact number of health behavior typologies was unknown, an explanatory approach was used. It started with the most parsimonious 1-class model and fitted successive models with increasing numbers of classes. Each latent class solution was replicated 20 times beginning at random starting values. This method included a close examination of item loadings and model fit indices for estimating latent classes [48]. The final number of classes was determined by the conceptual meaning and commonly used fit measures such as the Akaike Information Criterion (AIC), the Bayesian Information Criterion (BIC) and the value of entropy. The values of these indices for different class categories were shown in Table 2.

**Table 2:** Summary of Latent Class Model Identification and Statistics (*N* = 3267)

No .of classes	AIC	BIC	Entropy	Likelihood ratio chi-squared	P
1	38,847.4	38,908.3	–	3549.2	–
2	37,576.3	37,704.2	.663	2256.2	.00
3	37,064.4	37,259.4	.733	1722.3	.00
**4**	**36,788.7**	**37,050.6**	**.732**	**1424.5**	**.00**
5	37,833.8	38,165.2	.735	1273.2	.38

*Notes: AIC* Akaike’s information criterion, *BIC* Bayesian information criterion. Bolded row represents the identified model. The *P* value for 5 classes = 0.38, indicating we should accept 4 classes and reject 5 classes

When running LCA, the Stata software showed that convergence was not achieved when constructing 5 classes. Considering that the four-class solution provided the most conceptually coherent description of health lifestyles, the four-class solution was chosen as the most appropriate solution that represented health lifestyles among Chinese oldest-old. Since smaller values of AIC and BIC are better and the four-class model had both the smallest AIC and BIC (see Table 2), the statistical data proved that the four-class solution is preferred. As Table 2 presented, the entropy for the four-class model (0.732) was also beyond the criteria for good class separation cutoff point of 0.60 [49]. In order to further make sure that the correct number of classes was chosen, besides using Stata software, we also used Mplus to re-run the analyses and the results showed that all *P* values for 2,3,4 classes were 0, and the *P* value for 5 class was 0.38, indicating that we should reject choosing 5 class and accept 4 classes instead. Thus, the four-class solution was determined to be the best classification type to represent health lifestyles among Chinese oldest-old.

Snyder and Monroe [50] indicated two key assumptions for LCA. First, the conditional response probabilities for each individual in a latent class are assumed to be the same. Second, the assumption of conditional independence (also termed local independence). It asserts that within each class the indicators (i.e., variables) are independent of one another. Conditional independence enables us to express the probability of a particular pattern of responses conditioning on latent class only. Thus, we checked the conditional independence assumption when running the analyses. Muthen [51] contended that the conditional independence assumption was checked by examining the bivariate standardized residual z-scores in excess of |1.96| using the tech10 output option in Mplus. We followed the this rule and checked the conditional independence assumption. The Mplus results showed that such an assumption was met for most cases, except for the following pairs: (1)“sleep 8 hours and more” and “good quality sleep”; (2) “eat vegetable daily” and “eat fruit daily”; (3) participating active leisure activities” and “participating sedentary leisure activities“; (4) “participating active leisure activities” and “eta fruit daily” (Mplus results are available upon request). Although four pairs of variables did not meet the conditional independence assumption, we still decided to keep all variables for two mean reasons. First, there were numerous critiques of conditional independence. For instance, Hagenaars [52] asserted that “sometimes the constraints imposed by conditional independence are unrealistic”. Uebersax [53] stated that, in reality, some typologies can be better modeled by LCA when relaxing conditional independence. Reboussin, Ip, and Wolfson [54] contended that violating the local independence assumption was sometimes expected when examining certain types of behaviors, for example, drinking because each behavior would likely be related to others (e.g., binge drinking and having a hangover). Second, the variables that did not meet conditional independence assumptions were conceptually different. For example, eating fruit daily and eating vegetable daily represent different health lifestyles. Thus, we decided to relax the assumption of conditional independence assumption and allowed a few variables to be correlated.

Based on the results of LCA, the paper presented item response probabilities and shares for the analyzed sample for each class in Table 3. Such information clearly showed the four predominant health lifestyles among Chinese oldest-old and the percentage distribution of sample among the four classes. Meanwhile, the table also showed the percentage distribution of the respondents for each health behavior, which helped us to describe the patterns of the four classes that represented Chinese oldest-old’s health lifestyles.

**Table 3:** Item Response Probabilities for Health Lifestyle Indicators Used in Latent Class Analysis: Chinese Older Adults Aged 85–105, 2014

Variables	Class 1	Class 2	Class 3	Class 4
	(less healthy diet, not smoking, not drinking, poor sleep, low physical exercise& leisure activities; 32.4%)	(less healthy diet, not smoking, not drinking, good sleep, lowest physical exercise& leisure activities; 33.6%)	(consistent engagement in healthy behaviors; 21.9%)	(moderate diet, smoking and drinking, moderate sleep, moderate exercise and leisure activity engagement; 12.1%)
*Health lifestyle indicators*				
1) Eating fresh fruit almost everyday				
Yes	.038	.122	.314	.111
No	.962	.878	.686	.889
2) Eating fresh vegetables almost everyday				
Yes	.331	.467	.820	.554
No	.669	.533	.180	.446
3) Drinking tea almost everyday				
Yes	.095	.093	.313	.337
No	.905	.907	.687	.663
4) Smoking				
Yes	.099	.119	.299	.922
No	.901	.881	.701	.078
5) Drinking				
Yes	.089	.109	.190	.865
No	..911	.891	.810	.135
6) Good quality of sleep				
Good	.191	.932	.679	.661
Poor	.809	.068	.321	.339
7) Normally sleeps at least 8 h				
Yes	.231	.878	.505	.605
No	.769	.122	.495	.395
8) Exercising during the past & at present				
Yes	.126	.156	.558	.435
No	.874	.844	.442	.565
9) Participating in physical leisure activities almost daily				
Yes	.411	.331	.821	.573
No	.589	.669	.179	.427
10) Participating in sedentary leisure activities almost daily				
Yes	.234	.246	.897	.593
No	.766	.754	.103	.407

*Notes:* All variables are coded 1 = yes, 0 = no.

*Source*: Chinese Longitudinal Healthy Longevity Survey (CLHLS) 2014 data

### Descriptive and regression analyses

The analytical part started with descriptive analysis to report means and percentage distributions of all variables (see Table 1). Multiple regression models were then constructed to predict Chinese oldest-old’s subjective well-being on the basis of their health lifestyles, controlling for the respondent’s demographic and socioeconomic characteristics. Since the subjective well-being measures were coded as continuous variables, ordinary least squared (OLS) regressions were used to perform the analyses.

## Results

### Descriptive statistics

Descriptive statistical results for all variables were presented in Table 1. Among the 3416 respondents aged 85 to 105, there was a higher percentage share of rural than urban respondents in the sample (57.3 and 42.7%, respectively) and females outnumbered males (59.3% vs. 40.7%). The mean age of the sample was 93.1 with a standard deviation of 5.7. As to SES of the respondents, the average reported years of schooling among the oldest-old was 1.5with a standard deviation of 2.8.The mean household per capita income for the year before the survey was 15,832.8 RMB (which is equivalent to 2261.8 USD with 1 USD =7 RMB), with a standard deviation of 17,233.8. About 77.4% of the studied sample reported being hungry when going to bed in childhood. The average SRH score was 3.3 (between fair and good). About 34.8% of the respondents reported having ADL disabilities. Among the studied oldest-old, 19.8% of them reported living alone and 21.3% of them claimed that they experienced the death of a spouse during the past 3 years.

The health lifestyle patterns can be described as fairly healthy. The studied oldest-old seemed to be frequent fruit and vegetable eaters, with 13.5 and 49.1% of them eating fresh fruits and vegetables almost everyday. Tea was also preferred by some oldest-old and nearly one fifth of them had the habit of drinking tea almost everyday. About24.9 and 21.3% of the studied sample reported themselves as smokers and drinkers, respectively. For three fifth of the sample, sleeping was not an issue since they reported good sleep quality. A slightly over 55.0% of the sample had 8 or more hours of sleep each day. Considering the very old age of studied sample, the lifestyle of the respondents tended to be more sedentary than active. About 26.8% of them reported that they did physical exercise both before age 60 and at present when surveyed. The percentages of oldest-old who reported participating in at least one physical and sedentary type of leisure activities almost everyday were 48.4 and 41.7%, respectively.

As to the subjective well-being indices, findings showed that the average score of self-rated life was3.8 with a standard deviation of 0.78, meaning that overall, Chinese oldest-old rated their life as anywhere between “so so” and “good.” The average score for the positive feeling index was 7.2 with s standard deviation of 1.6. And the average score for the second negative feeling index was 7.0 with s standard deviation of 2.3.

### Health lifestyles among Chinese oldest-old

Based on the 4-class model (the best fitted latent class model), item probabilities for the four identified latent classes were estimated and the four predominant Chinese oldest-old’s healthy lifestyles (latent classes) and their share of the sample were presented in Table 3. Class 1 can be described as “less healthy diet, not smoking, not drinking, poor sleep, low engagement in physical exercise and leisure activities,” which contained 32.4% of the total sample. Sample in this class tended to be nonsmokers or nondrinkers (90.1 and 91.1%, respectively); they had moderate dietary patterns. Sample in this latent class experienced the highest level of inadequate nighttime sleep with those who had 8 h or more sleep everyday only counting 19.1% of the total sample. Respondents in this class also showed the lowest probability (12.6%) of doing physical exercise. Class 2 can be depicted as “less healthy diet, not smoking, not drinking, good sleep, lowest engagement in physical exercise and leisure activities.”This class consisted of 33.6% of all respondents, which had the highest percentage share of all sample among the four classes. Similar to class 1, this class showed moderate dietary pattern and sedentary lifestyles. But the major difference lied in the fact that Class 2 had relatively more smokers and drinkers and class 2 reported good sleep. Class 3, in contrast, can be defined as the “consistent engagement of healthy behaviors” group, which contained about 21.9% of all respondents. Chinese oldest-old in this class reported healthier lifestyles relative to most of their peers in other three classes across nearly all measures and domains. This group of the oldest-old reported the highest probabilities of having healthy dietary patterns. About 31.4, 82.0 and 31.3% of the oldest-olds in this class reported eating fresh fruits, vegetables and drinking tea almost everyday, respectively. The majority of them were not smokers or drinkers; they tended to have enough sleep (> = 8 h per day) and report good-quality sleep; there were relatively high percentages of them who participated in active and sedentary leisure activities and did physical exercises. Class 4 was delineated as “moderate diet, smoking and drinking, moderate sleep, moderate exercise and leisure activity engagement,” which comprised about12.1% of all respondents. The most significant feature of this group was having smoking and drinking problems. The respondents in this latent class reported moderate sleep and diet behaviors as well as activity patterns.

### The influence of health lifestyles on Chinese oldest-old’s subjective well-being

OLS regression models were constructed to predict the respondent’s subjective well-being. Table 4 presented OLS regression results when controlling for the respondent’s demographic and socioeconomic factors. The results presented in Model 1 showed that as compared to class 3(consistent engagement in healthy behaviors), the scores of evaluative subjective well-being, i.e., self-rated quality of life, for individuals in class 1 (less healthy diet, not smoking, not drinking, poor sleep, low physical exercise& leisure activities) were 0.21lower than the scores of those in class 3. The scores of self-rated life for oldest-old in class 2 (less healthy diet, not smoking, not drinking, good sleep, lowest physical exercise & leisure activities) and class 4 (moderate diet, smoking and drinking, moderate sleep, moderate exercise and leisure activity engagement) were 0.11 and 0.03 lower as compared to scores reported by members in class 3. These results suggested that less healthy lifestyles led to worse self-rated quality of life. Regarding the control variables, the results corroborated that an increasing age, higher family income, holding professional jobs before retirement and having better SRH had significantly positive effects on self-rated quality of life.

**Table 4:** OLS Regression of Subjective Well-Being on Health Lifestyle Latent Classes and Other Control Variables: Chinese Oldest-Old Aged 85–105, 2014

Variables	Self-Rated Life		Positive Feelings		Negative Feelings	
	Model 1		Model 2		Model 3	
	b	S.E.	b	S.E.	b	S.E.
***Health Lifestyle Latent Class*** (**Ref. = Class 3: Consistent engagement in healthy behaviors**)						
Class 1 (less healthy diet, not smoking, not drinking, poor sleep, low physical exercise & leisure activities)	−0.21***	0.06	−0.56***	0.13	0.87***	0.18
Class 2 (less healthy diet, not smoking, not drinking, good sleep, lowest physical exercise & leisure activities)	−0.11*	0.06	− 0.26***	0.13	0.43**	0.18
Class 4 (moderate diet, smoking and drinking, moderate sleep, moderate exercise and leisure activity engagement)	−0.03	0.06	−0.45**	0.14	0.45*	0.20
***Control Variables***						
Age	0.00***	0.00	0.01***	0.01	0.02	0.01
Sex (Ref. = female)	−0.06	0.04	0.06	0.10	−0.30*	0.14
Residence (Ref. = rural)	0.03	0.04	0.07	0.09	−0.22*	0.13
R’s years of schooling	0.01	0.01	0.04*	0.02	−0.06*	0.03
R’s natural logged per capita family income	0.09***	0.02	0.05*	0.02	0.04	0.06
R’s occupation before age 60 (Ref. = nonprofessional or non-administrative)	0.17*	0.09	−0.06	0.19	−0.05	0.27
R often when to bed hungry in childhood	−0.02	0.05	−0.26	0.10	0.06	0.14
R had ADL disability	0.07	0.04	−0.08	0.10	0.13	0.14
R’s spouse died since last survey year	0.09	0.05	−0.16	0.11	0.46***	0.15
R lived alone	0.03	0.17	−0.36	0.40	−0.18	0.53
R’s self-rated health	0.36***	0.02	0.56***	0.05	−0.77***	0.07
Constant	1.66***	0.39	4.40***	0.90	7.28***	1.27
N	1325		1167		1205	

*Notes:* * < .05, ** < .01, *** < .001. R represents the respondent. b: regression coefficient; *S.E.* standard error

*Source*: Chinese Longitudinal Healthy Longevity Survey (CLHLS) 2014 data

Regression results of using health lifestyle measures as well as control variables to predict positive and negative feelings of Chinese oldest-old were showed in Models 2 and 3, respectively. Model 2 showed that as compared to the reference group, class 3, scores of positive feelings for the oldest-old in classes 1, 2, and 4 decreased by 0.56, 0.26 and 0.45, respectively. In contrast, scores of negative feelings for the respondents in classes 1, 2 and 4 increased by 0.87, 0.43 and 0.45, respectively. The findings again highlighted that less healthy lifestyles stimulated negative feelings but lessened positive feelings of Chinese oldest-old. As to the covariates, an increasing age, higher SES and better SRH promoted positive feelings, whereas being rural, being females, having lower education and spouse’ death led to negative feelings in a significant manner. Those who self-rated their health better also tended to have lower negative feeling scores.

## Discussion

Through analyzing sample aged 85 to 105 from data of the CLHLS 2014 wave, this study examined health lifestyles among Chinese oldest-old and investigated how health lifestyles play a role in shaping the oldest-old’s mental health outcomes, such as subjective well-being. The research found four latent classes representing four predominant health lifestyles among studied Chinese oldest-old. The four classes included: class 1, a group with poor sleep issue (32.4% of total sample); class 2, a sedentary group (33.6% of all sample); class 3,“consistent engagement in healthy behavior” group (21.9% of total sample); and class 4, a group with smoking and drinking problems (12.1% of all sample). The percentage distributions of sample across the four latent classes suggested that individuals with poor sleep and sedentary lifestyles accounted for two thirds of the studied sample, which represented the most popular health lifestyles of Chinese oldest-old. Only 21.9% of the respondents demonstrated consistently healthy lifestyles.

Health lifestyles were found to be strongly associated with Chinese oldest-old’s subjective well-being, even after controlling for demographic features as well as individual and parental socioeconomic disadvantage. Consistent engagement in healthy behaviors linked to better self-rated life and positive feelings; whereas less healthy lifestyles were associated with higher negative feeling scores.

Previous research on the associations between lifestyle behaviors and health has largely focused on morbidity, mortality and disease prevention. For example, scholars examined how health lifestyle behaviors played a role in prevention of cardiovascular diseases [55] and cancer [56]. Findings of this research, however, underscored that more attention should be paid to relations between lifestyle behaviors and subjective well-being. With the trend of population aging and the oldest-old becoming a fast growing group in China, findings of this study reminded researchers and health professionals that practicing healthier lifestyles can be an effective way to improve Chinese oldest-old’s mental health status. Results of this research also echoed arguments of researchers that multiple health behavior change interventions outperformed single-behavior interventions in health promotion [57, 58]. Applying an integrative approach rather than individual health behavior perspective can be an effective way for health promotion. Findings based on analyzing the China data contained valuable implications to address disease prevention and health promotion related issues among older adults in other countries. Caregivers, clinicians and professionals may educate the elderly and their family to form healthier lifestyles for the purpose of healthy aging.

The significant impacts of the covariates on Chinese oldest-old’s subjective well-being also had important implications and were worth discussing. Age showed significantly positive effects on self-rated quality of life and positive feeling index among the oldest-old. The age effect could be a causality issue that those who have lived longer tended to have more positive attitudes towards life, which in turn, demonstrated a positive effect of age on subjective well-being. Higher SES and better SRH led to better subjective well-being, which was congruent with results of previous analyses. The urban and rural divide has been documented repeatedly in prior literature [59, 60], findings of this research also showed that rural respondents tended to report higher negative feeling scores as compared to their urban counterparts. The rural disadvantages showed among the sample of Chinese oldest-old reminded us that health promotion programs should lean more towards rural seniors. Unexpectedly, going to bed hungry did not show a significant effect on any of the oldest-old’s subjective well-being measures, suggesting that the cumulative disadvantage/advantage effects of parental SES on one’s subjective well-being may have disappeared in very old age. This pattern indicated a convergence trend of mental health differentials caused by one’s SES characteristics in later life. But even after controlling for these characteristics, the significant impact of health lifestyle measures on Chinese oldest-old’s health outcomes still existed. It again reminded us the need of taking into account health lifestyles in explaining the mental health disparities among the oldest-old.

## Strengths and limitations

The strength of this study was analyzing nationally representative sample to shed light on health lifestyle behaviors of Chinese oldest-old and explore the manner in which health lifestyles have shaped the oldest-old’s subjective well-being. Data included detailed measures assessing health lifestyle behaviors, wellbeing, physical and social functioning. Thus, conclusions drawn from this research can well represent general healthy lifestyle patterns of overall Chinese oldest-old population. Even with the strengths of the research, the study had several limitations. First and foremost, relying on examining the CLHLS dataset, the research was not able to exhaust all possible health lifestyle measures due to the availability of survey questions on health lifestyles. Some important health lifestyle indicators, such as vaccination injections, wearing seat belt, dental visits and et al. have not been included in this research. Secondly, evaluative well-being in this research was measured by means of a single-term question. Since some analyses have pointed out a significant association between sense of coherence and healthy lifestyle behaviors [6, 61], scholars may consider applying this subjective well-being component in future studies. In recent years, some researchers have pointed out the effect of “brain reserve” on preventing mental illnesses, which described some individuals having an increased “baseline adaptive neuroplasticity” that provides greater dynamic capacity for adjusting and remodeling cortical circuits to various stressors [62, 63]. Future study may consider taking “brain reserve” into account when study how health lifestyles affect mental health outcomes. Further, although findings of this study contained important implications to health promotion programs in other countries, the applicability of the results in other social contexts should still be tested considering the results were solely based on studying the Chinese sample. Lastly, regression models showed significant effects of health lifestyles on Chinese oldest-old’s subjective well-being. But since the dataset and the design of the research were cross-sectional in nature, the issue of causality has not been fully sorted out. It could be the case that better subjective well-being has caused healthier lifestyles. Future studies should consider using longitudinal datasets to further address the causality issue and unravel the interconnectedness of health lifestyles and elderly subjective well-being.

## Conclusion

Through analyzing data of the CLHLS 2014 wave, this research pointed out four latent classes representing four predominant health lifestyles among Chinese oldest-old. The four classes can be described as: class with poor sleep issue, a sedentary group, consistent engagement in healthy behavior group, and the group with smoking and drinking problems. Overall, the majority of the Chinese oldest-old had sedentary lifestyles with poor sleep issues; only 21.9% of the respondents showed consistently healthy lifestyles. The study concluded that healthier lifestyles led to better self-rated quality of life and positive feelings among Chinese oldest-old. Less healthy lifestyles, in contrast, were more likely to cause negative feelings. This is one of the first studies that assessed Chinese oldest-old’s health lifestyles and their subjective well-being. Findings of this research contained important implications in terms of enhancing older adults’ subjective well-being as well as mental health status in other world countries. Older adults can tackle healthier lifestyle behaviors in their daily lives to reduce the risk of mental health problems. Practicing healthy lifestyles should be integrated in programs for mental health promotion.

## Supplementary Information

**Additional file 1:** Appendix Scale Items and Alpha Coefficients.

## Data Availability

This article is based on a publicly available dataset derived from the Chinese Longitudinal Healthy Longevity Survey (CLHLS). The dataset can be obtained after sending a data user agreement to the data team.

## Abbreviations

ADL: Activities of daily living
SRH: Self-rated health
SES: Socioeconomic status
