# Supplementary material for: Health lifestyles and Chinese oldest-old’s subjective well-being—evidence from a latent class analysis
Source: BMC Geriatr. 2021 Mar 24;21:206. doi: 10.1186/s12877-021-02121-0 (PMC7992951; doi:10.1186/s12877-021-02121-0)
Supplement: Supplementary file 1 — Additional file 1: Appendix Scale Items and Alpha Coefficients. [file 12877_2021_2121_MOESM1_ESM.docx]

| **Appendix. Scale Items and Alpha Coefficients** | | |
| --- | --- | --- |
| Variables | Individual Items | Alpha Values |
| Evaluative | Q1: How do you rate your life at present? |  |
|  |  |  |
| Positive feelings | Q2: Do you always look on the bright side of things? | 0.51 |
|  | Q3: Do you feel happy as younger? |  |
|  |  |  |
| Negative feelings | Q4: Do you feel fearful or anxious? | 0.63 |
|  | Q5: Do you feel lonely and isolated? |  |
|  | Q6: Do you feel useless as age? |  |
